# Correction: Data-driven modeling predicts gene regulatory network dynamics during the differentiation of multipotential hematopoietic progenitors

**DOI:** 10.1371/journal.pcbi.1013823

**Published:** 2025-12-19

**Authors:** Joanna E. Handzlik, Manu

In the Funding section, one of the grant number from the National Science Foundation is missing. The additional grant number is: #1942471.
