# Supplementary figures and images for: Data-driven modeling predicts gene regulatory network dynamics during the differentiation of multipotential hematopoietic progenitors
Source: PLoS Comput Biol. 2022 Jan 14;18(1):e1009779. doi: 10.1371/journal.pcbi.1009779 (PMC8794271; doi:10.1371/journal.pcbi.1009779)

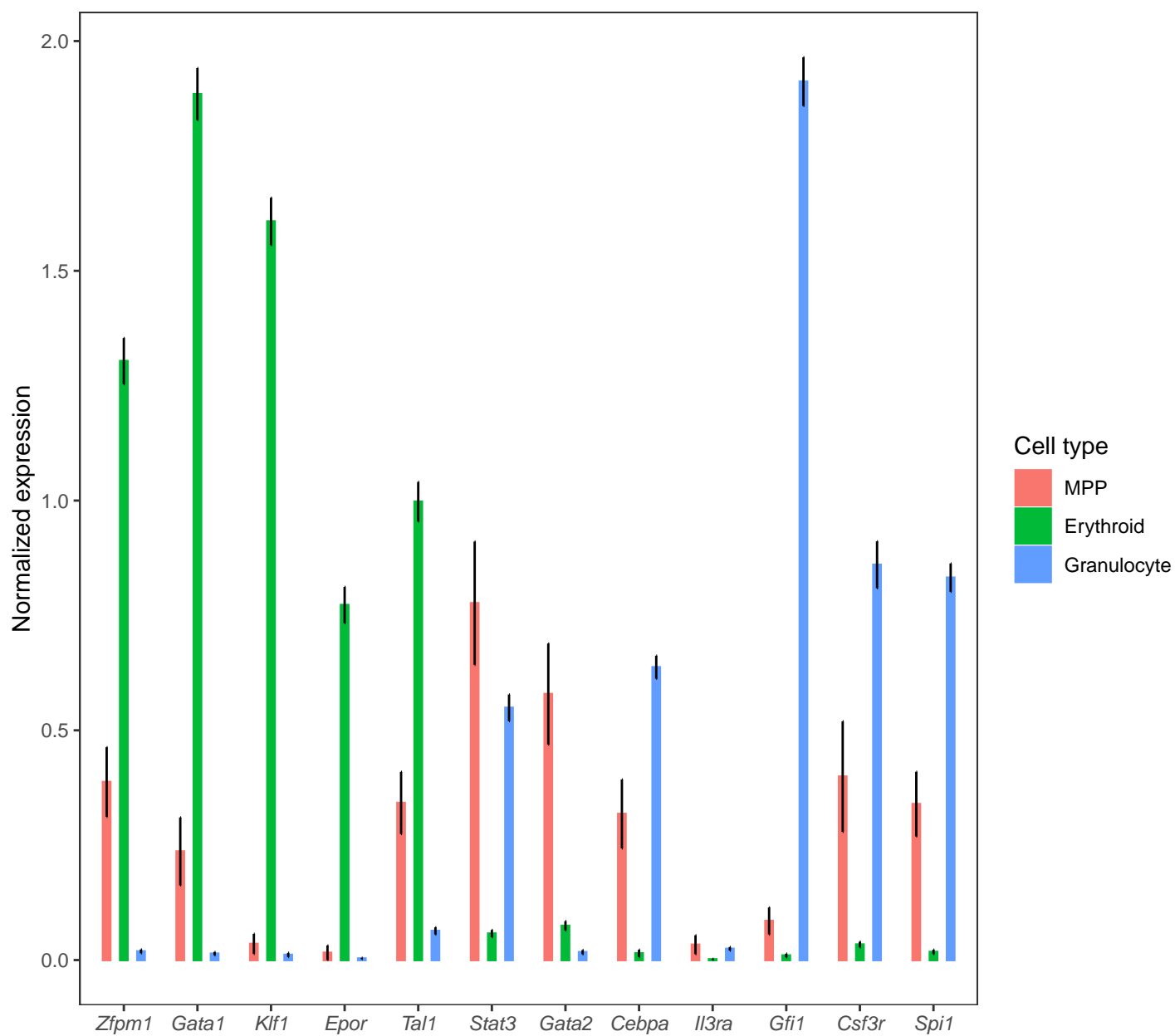

Supplement: S1 Fig — The average expression in MPPs, erythroid progenitors, and granulocytic progenitors is shown for the modeled genes. Erythroid and granulocytic progenitors were identified as having a PBA erythroid and granulocytic probability (see Materials and methods) greater than 0.9 respectively. MPPs were identified as cells having a low PBA probability (< 0.2) of belonging to any lineage. Error bars show the standard error of the mean. (PDF) [file pcbi.1009779.s001.pdf]

RMS (log10)

10.0  
1.0  
0.1

Permuted  
models

Unpermuted  
models

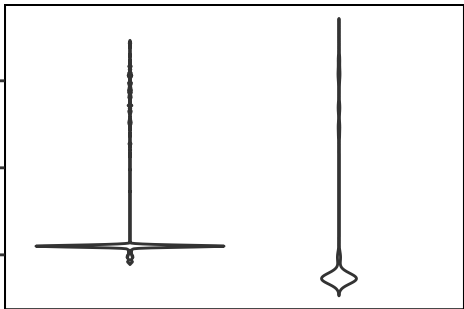

Supplement: S2 Fig — The distributions of the RMS scores of gene circuits trained on real data (Unpermuted models) or on randomized synthetic data (Permuted models) are shown as violin plots. The scores were compared using the Wilcoxon ranksum test with continuity correction (p = 3.8 × 10^−8^). (PDF) [file pcbi.1009779.s002.pdf]

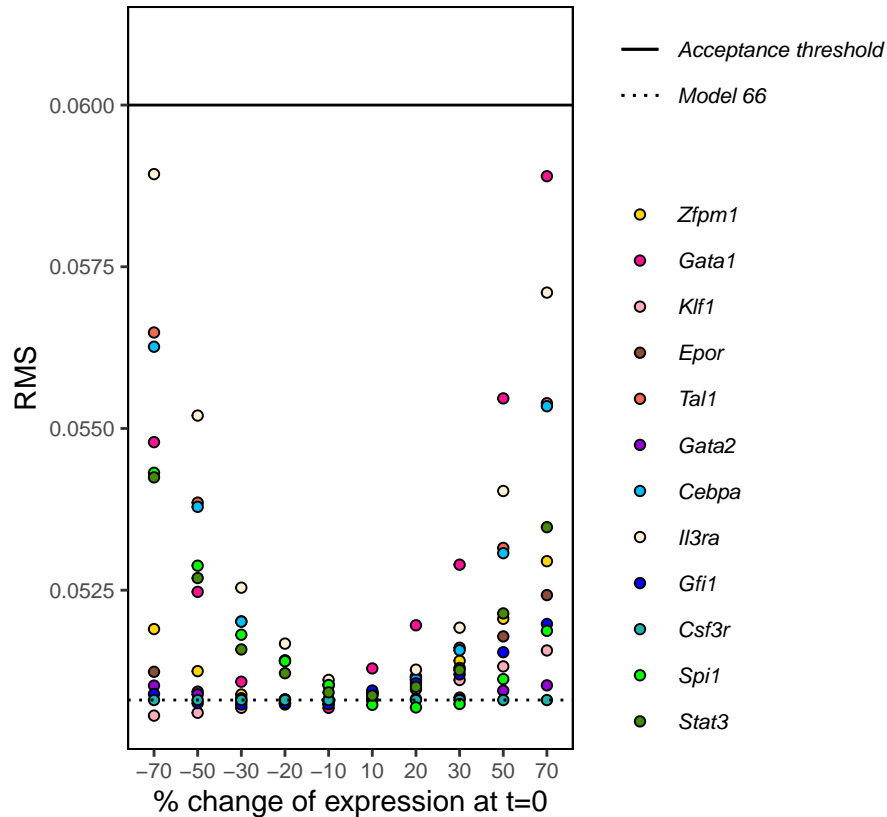

Supplement: S3 Fig — Model 66 was run with the initial conditions perturbed one gene at a time. The x-axis is the magnitude of the perturbation. The y-axis is the RMS. The perturbed gene is indicated by the color of the points. The dotted line is the RMS of model 66 and the black line is the goodness-of-fit threshold RMS. (PDF) [file pcbi.1009779.s003.pdf]

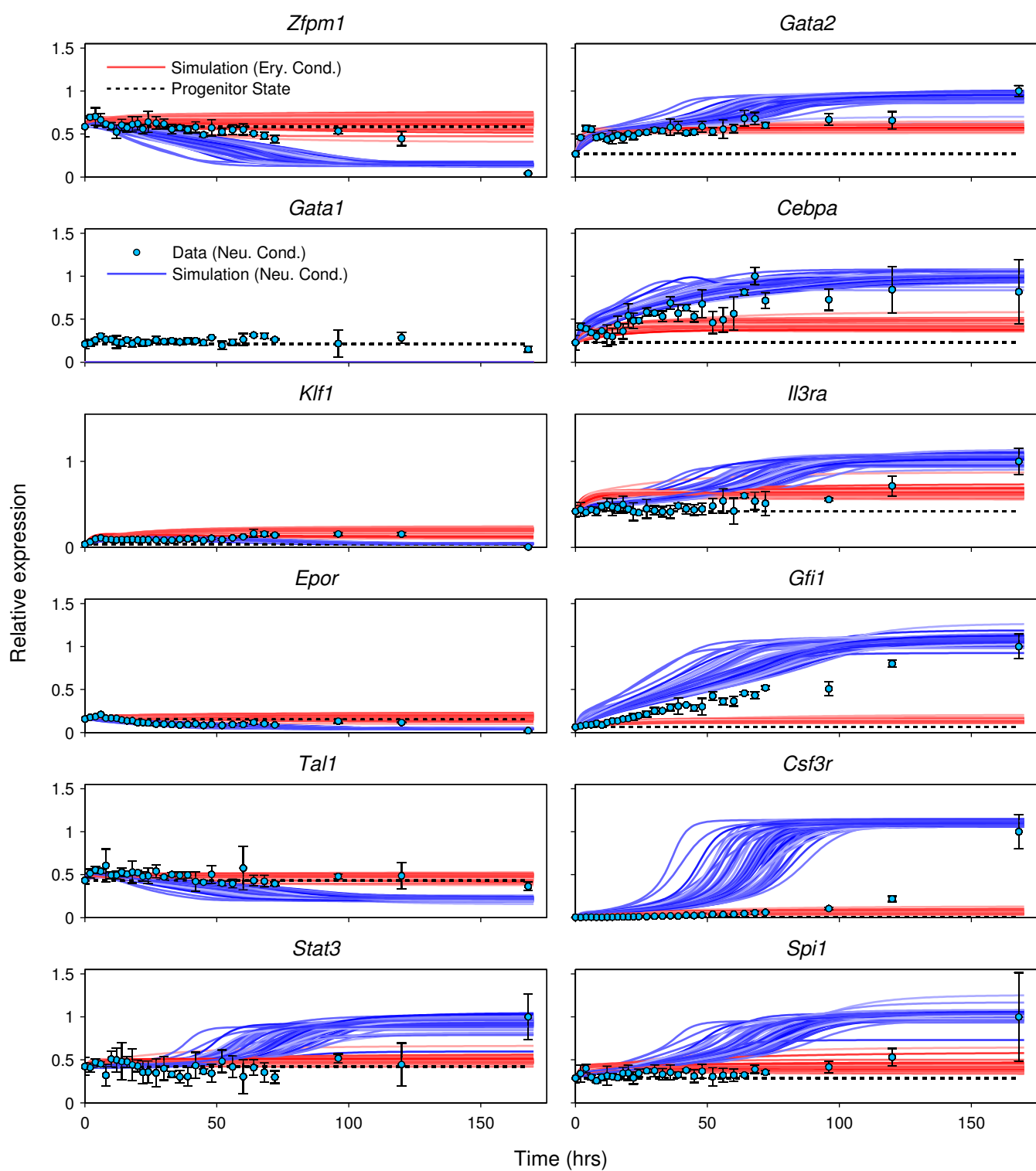

Supplement: S4 Fig — Gata1 knockout was simulated in all 71 models that met the goodness-of-fit criteria. Their output is plotted as lines. The symbols and colors are the same as Fig 1. (PDF) [file pcbi.1009779.s004.pdf]
