# Supplementary material for: Data-driven modeling predicts gene regulatory network dynamics during the differentiation of multipotential hematopoietic progenitors
Source: PLoS Comput Biol. 2022 Jan 14;18(1):e1009779. doi: 10.1371/journal.pcbi.1009779 (PMC8794271; doi:10.1371/journal.pcbi.1009779)
Supplement: Attachment — Submitted filename: grn_ery_neu_response_to_reviewers.pdf [file pcbi.1009779.s007.pdf]

## Responses to reviewer comments on manuscript #PCOMPBIOL-D-21-01078

**Overview:** We were happy to see the generally positive reviews and thank the reviewers for their helpful and insightful comments. We have revised the manuscript to address the reviewers' points by performing additional simulations, adding two supplementary figures, and including additional explanations. We carried out additional simulations to test the robustness of the model to variations in initial conditions (reviewer #1, point 2a) and the results are included as a new supplementary figure (Fig. S3). We checked the expression of the modeled genes in primary cells as suggested by reviewer #2 (point 3) and have added a supplementary figure (Fig. S4) showing those patterns. We believe that the manuscript is much improved as a result. A point by point description of the changes follows.

### Major points

### Reviewer 1

1. In a two-part point (#2), reviewer 1 expressed the concern that gene circuits model the average behavior of the population and that the inferences might change if cell-to-cell variability in gene expression or population heterogeneity were to be taken into account. First:

   > 2. a) First, in this model, it's assumed that if two transcription factors are both expressed at certain levels in the population, then they are expressed in the same cells. However, they may not be - the Gata2 expression may be in different cells from the Gfi1 expression, for example. While a different kind of model would be needed to deal with probabilistic aspects of heterogeneous population responses, it would be good to acknowledge that this is an issue. Ideally, the authors might calculate how robust their top models would be in a few examples where the population was generating some reasonable, specific mixtures as intermediates.

   We agree with the reviewer that a full stochastic treatment is out of the scope of this manuscript and would require both an entirely new modeling framework and an entirely new single-cell time-resolved dataset. However, we have followed the reviewer's suggestion to check the robustness of the model to variations in initial conditions. We varied the initial concentrations of the gene products one at a time within $\pm 70\%$ of their original values and determined how well the pertubed models agreed with data by computing the root mean square (RMS) score. As one would expect RMS increases with the magnitude of the perturbation, but remains below 0.06, the goodness-of-fit criteria we utilized to identify acceptable model fits. This result, included in the revised manuscript as Figure S3, shows that the inferred gene circuit is robust to variations in initial conditions and produces the observed cell fates even with initial conditions considerably different from those in the bulk gene expression data.

   We have also included a paragraph on p. 26 acknowledging the limitations of a deterministic modeling framework.

   We would like to mention that single-cell gene expression analysis supports the co-expression of genes from multiple lineages at a single-cell level in the EML progenitors (Pina et al., 2012) and the scenario

of the observed patterns arising from clearly separable subpopulations with distinct gene expression programs is unlikely.

2. In the second part of point #2 the reviewer expresses the concern that the regulatory inferences could be erroneous since the averaged gene expression patterns could be produced by changes in the distribution of the subpopulations of a heterogeneous mixture rather than by gene regulation happening at a single-cell level.

> 2. b) Second, related to point 1, the goal here is to infer the true underlying gene network structure on the basis of the gene expression signatures that predominate. But some gene network features might be missed if they led the cells to dead ends in the culture systems used by May et al. Are erythroid genes unexpressed in the neutrophil conditions because they are actively repressed, or because any cells taking the erythroid pathway are dying out? Also, if Gata1- nonexpressing cells were to upregulate PU.1 prematurely, would this drive them toward a macrophage fate that makes them proliferate less, in both of the tested sets of culture conditions? Is this an alternative explanation for why earlier PU.1 upregulation is not seen? It would be helpful if the authors discussed the possibility that pathway-specific cell-survival constraints could also be "trimming" the outputs from the gene network. Would this reduce the need for direct repression operating early in the network?

We have discussed this possibility in paragraph 2 on p. 26 as requested. Although we cannot rule out the possibility completely and a definitive answer requires single-cell data and stochastic models, we argue that most of the inferred connections have a sound basis. A large proportion of inferred connections agreed with genetic and biochemical manipulations that are not confounded by heterogeneity (Fig. 4 and Table S2). Regarding the specific examples of PU.1 and Gata1 that the reviewer mentions, Hoppe et al. (2016) have shown that PU.1 is upregulated at a single-cell level at the late stage of myeloid differentiation, while Gata1 is upregulated at a single-cell level during erythroid differentiation. Rieger et al. (2009); Mossadegh-Keller et al. (2013) have shown lineage commitment occurring at a single cell in the absence of significant cell death.

3. Reviewer #1 would like us to clarify how the amount/concentration of regulator impacts the expression of the target: monotonic and gradual manner, monotonic and sharp manner, or in a non-monotonic manner.

> 3. Please, be clearer about how the model takes into account possible dose-dependent changes in regulator impact. This is important, since there is much published evidence for different developmental effects of PU.1, at least, depending on its level of activity, either absolutely or relative to other factors. One possibility is that these factors always act in the same direction on a given target, and in a continuous-valued way as a function of dosage. Another is that these factors always act in the same direction on the target, but have a sharp dosage threshold below which they have no effect. A third possibility would be that a factor might switch its effect from positive at a low level of expression to inhibitory at a high level of expression (e.g. by binding to different sites as its concentration rises). How many of these possibilities are taken into account in the model construction?

We have explained in greater detail in paragraph 2 on p. 28. Briefly, dose dependence works in the first two ways suggesed by the reviewer but not in the third. Whether a regulator is an activator or

repressor does not change with its expression or concentration. Although such mechanisms have been hypothesized for some regulators, we purposely do not allow them since it increases the degrees of freedom and the risk of overfitting in the model.

4. The reviewer asks whether the famous switch from Gata2 to Gata1 during erythroid differentiation is explicitly represented in the model and, if not, whether this may explain the inability of the model to infer Gata2-related parameters.

> 4. It was surprising not to see more explicit use of evidence for the relationship between Gata1 and Gata2. The Zfpm1-dependent "Gata switch" (Gata2+Zfpm1 first activating Gata1, but Gata1+Zfpm1 then repressing Gata2) is considerably discussed in the literature (Orkin and Bresnick groups) but does not seem to emerge from the model. Could the lack of the Gata switch explain some of the problems with predicting Gata2 levels?

Our modeling approach does not seek to prescribe any particular interaction in the GRN but attempts to infer the interactions from time series data. This was discussed in several places in the manuscript (paragraph 3 on p. 4; paragraph 3 on p. 21, paragraph 3 on p. 13, and Section 4.3). We could ask whether the model inferred repression of Gata2 by Gata1, and the answer is that the model correctly inferred the sign of the interaction (Fig. 4; Gata2 panel). The model also inferred correctly that Gata2 represses Gata1 (Fig. 4; Gata1 panel). So even though we did not hand-craft these interactions into the model, training with the time series data did infer at least the type of interactions. It is possible that the strength of these interactions was not inferred correctly or that how Gata2 regulates genes other than Gata1 were not inferred correctly, resulting in incorrect predictions of Gata2 knockdown. These possibilities have been discussed in paragraph 4 on p. 24 of the manuscript.

5. Reviewer #1 correctly deduces that the model does not require pairs of factors, such as Fog1 (Zfpm1) and Gata1 to only act when both are expressed and inquires whether the model requires Gata1 to be expressed in order for Fog1 to act.

> 5. Fig. 5 does not seem show any evidence for transcription factors acting obligatorily in pairwise combinations in order to generate specific outcomes. The structure of equation 1 seems to require measuring each factorâĂŹs effect on the other genes alone, one factor at a time. One of the major activities in the early stages of the erythroid pathway in Fig. 5 seems to be Zfpm1 by itself. However, Zfpm1 presumably cannot bind DNA by itself. Also, there is considerable evidence that Gata factors with Zfpm1 function differently from Gata factors without Zfpm1 (e.g. work from Crispino). Does the model take into account the inability of Zfpm1 to affect gene expression unless it is binding to a Gata factor?

Reviewer #1 is correct that the structure of Equation 1 does not impose any pairwise constraints on the actions of the regulators. Once again, this modeling choice was made in order to guard against overfitting the model, which would reduce its predictive power. Potential obligate co-regulation of a target by pairs of regulators can be allowed by including pairwise product terms in Equation 1, but this would significantly increase the degrees of freedom, and hence the potential for overfitting, in the model. The explicit representation of co-regulation by a Fog1/Gata1 complex may be dispensible in hematopoietic tissues where Fog1 and Gata factors are always co-expressed but may be necessary in non-hematopoietic tissues where Fog1 is expressed while Gata1 is not. The lack of this interaction

may limit the generality of the present model but we hope that reviewer will agree that the construction of more biochemically explicit but comprehensive models should be the subject of future models.

We have acknowledged this limitation in the Discussion section in paragraph 2 on p. 25.

6. Reviewer #1 points out that FDCP-mix cells are multipotential as are hematopoietic progenitor and wonders whether any of the models not analyzed in detail simulate lineages other than erythrocytes and neutrophils.

> 1. It is somewhat troubling that the model is framed as though the normal hematopoietic decision is between erythrocyte fate and granulocyte fate. That is clearly not a direct binary choice in the Tusi et al 2018 data used in the later part of the paper, where the non-erythroid branch leads to sub-branches for lymphocytes, dendritic cells, and macrophages before the long neutrophil spur emerges. The granulocyte-macrophage decision network has been dissected at the single-cell level by Olsson...Grimes 2016, for example. Even with FDCP-mix cells, the original Spooncer 1986 paper shows macrophage emergence as well as erythroid and neutrophil production. Did any of the 70/71 models that were not shown in depth in the paper incorporate more alternative lineage options?

Which potential FDCP-mix lineage outputs the models are capable of producing is constrained by their construction and the nature of data-driven modeling. In order for a model to simulate a particular lineage, its construction must include the key genes expressed in that lineage. Our models, and nearly all systems biology models, have to be trained on data in order to determine the values of free parameters. Taken together these two aspects of data-driven modeling imply that we can only include those combinations of genes and lineages for which training data are available. Our models were trained on genome-wide gene expression data from the differentiation of FDCP-mix cells towards the erythrocyte (low IL3, Epo, and hemin) or neutrophil fates (GCSF and SCF) and therefore cannot simulate other lineages in their present realization. The model can simulate conditions that it was not trained on, such as genetic perturbations (Fig. 2 and 3), but this capability does not extend to other lineages. The 71 models were generated to check whether quantitative differences in the values of the parameters impact the overall behavior of the model and hence ensure that the analyzed model is representative and not unusual in some manner. All these models are however limited to the lineages that they were trained on for the reasons discussed above.

This does not preclude the construction of gene circuits having multilineage capability in the future. The main missing piece is genome-wide gene expression time series data from other lineages—the subject of current projects in the lab. This was mentioned in the last sentence of the discussion.

**Reviewer 2**

1. The reviewer advised us to check the expression patterns of the modeled genes in primary cells.

> (3) While FDCP-mix cells have been useful for several studies, they are not the same as primary, multi-lineage progenitor cells. Thus, it would be wise to insure that the training data used (from the May et al. study in FDCP-mix cells) are consonant with data from primary cells. For example, the data summarized in Fig. 1 show RNA levels for Gata2 increasing

> during neutrophil differentiation, but my examination of multiple RNA-seq datasets across hematopoietic differentiation does not reveal that accumulation of GATA2 in primary neutrophils or monocytes.

We have checked expression patterns of the modeled genes in the mouse bone marrow dataset of Tusi et al. (2018) and included them in a supplementary figure (Fig. S4). The expression in MPPs, erythrocyte and granulocyte progenitors largely agree with the FDCP-mix dataset at a qualitative level with the exception of *Gata2*, which, as the reviewer notes, is not upregulated in the granulocytic lineage.

This further highlights that the biggest failure of the model, in inferring *Gata2*-related parameters and predicting the consequences of *Gata2* knockdown, has its origins in the training dataset. We have noted this in paragraph 2 on p. 11 and paragraph 3 on p. 24. We thank the reviewer for pointing this out.

2. The reviewer would like us to explain why more genes were not included in the model and also whether the major conclusions would differ if more genes were included or these genes were replaced by others.

> (2) What was the logic for including only the 12 genes in the circuits and networks? Multiple lines of investigation support important roles for the 12 genes in blood cell differentiation, but those prior studies also implicate other genes. For instance, roles for Gfi1b, Meis1, Fli1 (and genes encoding other ETS factors in addition to PU.1), Runx1, other genes. Was there a computational limit on the number of genes that could be included? Were the major observations (e.g. highly interconnected networks, inferred late role for Spi1) robust to the inclusion of more genes or replacement with other genes?

The logic of including these 12 genes was explained in the first paragraph of the results. We have added a paragraph explaining why the models were limited to 12 genes (paragraph 2 on p. 6). The reviewer correctly guesses that there are computational limits; one set of fits took 5 days running on 100 CPUs of a modern HPC system and it took many iterations to find good optimization conditions. The computational effort wasn't the most important consideration though. Increasing the number of genes increases the number of free parameters, which makes overfitting more likely. We took the conservative approach of having three-fold excess of datapoints compared to free parameters and the success of the model in predicting genetic perturbations seems to support our decision.

The reviewer also asks whether the major conclusions are robust with respect to the number and/or choice of genes in the model. Constructing alternative models with different genes or more genes would be a form of validation. We have already performed several types of validation. First, we have scrambled the gene expression patterns (Fig. S1), which is equivalent to replacing the genes with non-biological genes. Second, we have painstakingly compared the interconnections to the literature (Fig. 2 and Table S2). Third, we have made genetic perturbations and compared against empirical data (Fig. 2 and 3). Fourth, we have checked the model inferences about the order of Spi1/Cebpa/Gfi1 expression in an independent *in vivo* dataset (Fig. 7).

Checking whether the main conclusions are preserved when changing the number or identity of genes is not the best form of validation. Genes are unique and not interchangeable; if a model conclusion changes when we swap out a gene, is it because the conclusion is not robust or because the gene was essential to the function of the GRN? Similarly, if we increase the number of genes and the

models perform differently, is it because the new genes are playing essential functions or because there are more parameters and it is easier to fit the model? Furthermore, there is a combinatorial explosion of possibilities as you start to increase the number of genes in the model, so that in addition to the difficulty of interpreting the results, the computational expense makes this sort of validation impractical if not impossible. We considered validation against independent experimental results much more useful and hence prioritized it over other forms.

3. Reviewer #2 asks us to evaluate May *et al.*'s 3-gene model of Gata1, Gata2, and PU.1 (May et al., 2013), (apparently) an evolution of the bistable switch model, using gene circuits.

   > (4) The manuscript states the bistable switch model in the simplest terms, but in the May et al. paper (that is the source of the RNA-seq data used in this manuscript)s, the model has evolved to a three-way model with GATA1, GATA2, and PU.1. Importantly, the May et al. paper has ChIP-seq data that supports co-occupancy by GATA2 and PU.1 in the "multilineage progenitor cells". The manuscript under review should evaluate this more advanced, 3- protein model by the gene circuits.

   The model in May et al. (2013), although including Gata1 and PU.1, is not a bistable switch model (Huang et al., 2007; Laslo et al., 2006) since it only simulates gene expression during erythroid differentiation (Figure 6C of May *et al.*). In order to be a "switch" the model must simulate at least two alternative fates.

   The Gata1-Gata2-PU.1 network is already a subset of our 12-gene gene circuit and we can compare it to May *et al.*'s model. The main conclusion that May *et al.* reached with their model was that Gata2 represses PU.1 (Figure 6D of their paper). Our gene circuit has inferred the same interconnection (Figure 4, *Spi1* panel). Our fits are, of course, superior since we match not just the erythroid differentiation data but the neutrophil differentiation data as well.

   Regarding the use of ChIP data, May *et al.* increase or decrease the strength of kinetic parameters exponentially in time if the height of the ChIP peak increases or decreases respectively. Adjusting the kinetic parameters in this *ad hoc* manner doesn't have any biophysical or biochemical basis. The net effect of modeling kinetic parameters like this is to "double count" the concentration of the regulator. The height of a ChIP peak presumably reflects the occupancy of the TF, which is determined by the TF concentration. Modulating a kinetic parameter in time and then multiplying it to the concentration is to account for the change in the concentration twice.

   Overall our model 1) is more comprehensive because it includes more genes, 2) actually models the cell-fate decision by accurately simulating both erythroid and neutrophil conditions, and 3) simulates all the timepoints instead of stopping at day 5 as the May *et al.* model does.

4. The reviewer questions whether the Tusi et al. (2018) dataset disproves the bistable switch model since Spi1 is expressed in the early stages.

   > (5) The manuscript includes an analysis of single cell RNA-seq to evaluate their major conclusions about determinants of neutrophil cell fate. However, it is not clear that those data argue against the bistable switch model with Spi1 (PU.1) playing an early role. The single cell RNA data shows that the Spi1 transcript levels reach a peak after the peaks of Cebpa and Gfi1 (Fig. 7), supporting the conclusions in the manuscript. However, those data also show that Spi1 transcripts are present, even higher than Gfi1, in the early stages.

> The fact that Spi1 transcripts are present does support the frequently stated binary (bistable switch) model for antagonistic repression between PU.1 (SPI1) and GATA1.

We would like to clarify that our model or analysis does not challenge antagonistic repression between Gata1 and PU.1. Our model infers that Gata1 and PU.1 repress each other and autoactivate their own expression (Fig. 4) which is precisely how previous bistable switch models have been formulated (Huang et al., 2007). We also took care to predict the consequences of Gata1 or PU.1 knockdown with the model, which performs as one would expect if there were mutual repression between Gata1 and PU.1.

Gata1-PU.1 bistable switch models (Huang et al., 2007; Chickarmane et al., 2009) always include an unknown signal "X" (hypothesized to derive from cytokine signals) that triggers differentiation. Our model differs from those models in two main ways pertinent to this question. First, our model has many more genes and was thus able to infer that "X" is Cebpa and/or Gfi1. Secondly, in some implementations (Huang et al., 2007) of the bistable switch model, Gata1 and PU.1 are expressed at low levels in progenitors and lineage-choice is initiated by fluctuations in the initial concentrations of Gata1 and PU.1. In our model, cell-fate choice is driven by the effects of the cytokines and not by the initial concentrations of Gata1 and PU.1.

We have reinforced the point that our model does include and rely on mutual repression between Gata1 and PU.1 in paragraph 3 on p. 21 and the last sentence of paragraph 2 on p. 22.

We also agree with the reviewer that the scRNA-Seq data on their own do not disprove or argue against the bistable switch model. Our conclusions, however, don't rely on PU.1 being completely off and then turning on later. In fact, both undifferentiated FDCP-mix cells (Fig. 1) and HSCs (Hoppe et al., 2016) express PU.1. PU.1 expression increases during myeloid differentiation in the FDCP-mix system, Tusi et al. (2018) scRNA-Seq data, and in the live-cell tracking carried out by Hoppe et al. (2016, ; Fig. 4b). It is not mere PU.1 expression that distinguishes between progenitors and granulocytes but that PU.1 is expressed at a higher level in the latter. Our analysis thus focusses on PU.1 upregulation, which lags that of Cebpa and Gfi in both FDCP-mix and scRNA-Seq data, and not the actual level. We have made this point clearer in paragraph 2 on p. 19 and paragraph 2 on p. 20.

We would like to clarify though that the patterns in Fig. 7B are normalized (as noted in the figure legend) so that the maximum expression is set to 1. Although the absolute expression of a gene is not informative (since it could differ from protein expression level and the protein activity could be very different between genes), Gfi1 is, in fact, expressed more robustly than Spi1. This is apparent in Fig. 7D.

5. The reviewer asks why Spi1 and GCSF are treated differently in our reasoning.

> (6) Text in the Discussion implicates GCSF signaling as a key instruction for differentiation to granulocytes. The gene encoding the receptor for GSCF, CSF3R, is part of the GRN. However, its expression rises dramatically late in the differentiation to neutrophils. This is the same kind of data that the authors use to argue that the Spi1 gene cannot be an early determinant of differentiation. The manuscript should explain this apparent discrepancy and utilize the same criteria for inference about early versus late-acting factors.

In the discussion, we reasoned that GCSF signaling is the "ultimate" cause of neutrophil differentiation in FDCP-mix cells because treating them with GCSF causes neutrophil differentiation. This fact

stands on its own and is true irrespective of the temporal progression of the expression of the receptor. The asymmetry is caused not by the expression of the receptor but by the presence of the GCSF ligand in the culture medium, which causes the activation of STAT3, C/EBP$\alpha$, and other TFs (Jack et al., 2009).

In contrast to GCSF signaling, which does not rely strongly on the amount of receptor, PU.1 is thought to act in a concentration-dependent manner (DeKoter and Singh, 2000; Dahl et al., 2003), which is an explicit assumption of the bistable-switch model (Huang et al., 2007; Chickarmane et al., 2009). Since PU.1 activity is tied to its concentration, late upregulation puts its status as a causal driver of neutrophil differentiation in doubt. We have added a sentence clarifying this in paragraph 4 on p. 17.

All of this applies to FDCP-mix cells but we do not know if GCSF signaling is the trigger *in vivo*. We were already careful in saying that "... we propose that ..." GCSF signaling causes neutrophil differentiation *in vivo*. We have changed the wording to "... we speculate ..." to make it absolutely clear that this is not a statement of fact.

6. (1) The construction and products of the gene circuits need to be explained specifically and clearly. It is not clear how the ordinary differential equations (ODEs) obtained by training gene circuits differ from those that led to the bistable switch models espoused by May, Enver and others. The manuscript does state that prior work with ODEs was limited to a small number of genes. What are the fundamental differences between the described and prior work? Exactly what is the output of the gene circuit analysis? A table of interaction coefficients is provided, but it seems that many other parameters are modeled. Can the resulting models be used by others in further analyses?

We have reworked paragraph 3 on p. 21 to discuss the differences between previous bistable switch models and gene circuits in detail.

We're not sure what the reviewer means by the "output of the gene circuit analysis" since all figures except the last are outputs of the gene circuit analysis. As described in the Methods section (Sections 4.1 and 4.3), the primary result of the training are the parameters of Equation 1: genetic interconnectivity ($T_{ij}$), cytokine regulation strengths ($c^l$), sigmoid thresholds ($h_i$), the maximum synthesis rates ($R_i$), and the degradation rates ($\lambda_i$). For the 12 gene GRN there are 192 parameters per model. We have provided all 192 values for all the 71 models that passed our quality criteria, in a 71 $\times$ 192 element table (Table S1). There are no other parameters in the model. In principle, this is all anyone needs to know to create the models by implementing Equation 1. However, we have provided the code and the input files on GitHub (in the Data Availability section) so that others may run the model(s) for further analyses.

7. (8) The manuscript needs to state some of the conclusions more carefully. For example, on page 20: "We showed that this model is biologically accurate by correctly predicting the consequences of genetic perturbations..." would be more appropriately stated as "The correct predictions of the consequences of genetic perturbations... support the accuracy of the model". At the same time, the authors should state how the inaccurate predictions of the model should be evaluated in terms of overall utility of the models.

We have implemented the suggested language. The limitations of the model and inaccurate predictions have been discussed in detail starting from paragraph 3 on p. 24 up to paragraph 2 on p. 26. We have added a sentence to the end of paragraph 3 on p. 24 explaining that the model should still be useful provided we are careful about inferences related to Gata2.

**Other points**

**Reviewer 1**

1. 6. In Table S1, the headings are misaligned with the columns of parameter values - please check and fix!

   Thanks for catching that. Fixed.

2. 1. The conclusion that cells can go far in the neutrophil lineage with minimal PU.1 expression fits the evidence that complete PU.1 knockouts do generate immature granulocyte-like cells, but not lymphocytes or macrophages (Dakic...Nutt 2005). This experimental observation is in good harmony with the predictions of the model and should be noted.

   Thanks for the tip!

3. 2. There is evidence in the literature using sensitive single-molecule detection that PU.1 and GATA-2 are very frequently coexpressed in individual myelo-erythroid progenitors (Wheat et al 2020 Nature). Therefore, it is misleading to imply, as on p. 3 and p. 19, that PU.1 is not expressed in granulocyte-lineage differentiation at all until its late increase in expression.

   We didn't mean that. "...Cebpa and Gfi expression precedes that of PU.1..." has been changed to "...Cebpa and Gfi1 expression precedes the neutrophil-specific upregulation of PU.1..." on both pages.

4. 3. The references should be re-checked. On p. 9, is the citation to ref 45 as meant? Also, there are some references duplicated under different numbers: 39 the same as 67, 44 the same as 63.

   Fixed. Thanks!

5. 4. Several times throughout the manuscript, "its" (meaning, belonging to it) is spelled "it's".

   Fixed. Thanks!

6. Please provide tick marks for the right-hand plots in Fig. 2. Are these "relative expression" units the same as the ones in Fig. 1?

   We're not sure what is meant here. The panels on the right in Fig. 2 have both X- and Y-tick marks - the same as the panels on the right in Fig. 1.

   Yes, the units are the same and Fig. 1 and 2 may be compared directly.

**Reviewer 2**

1. (7) The text is overly long, and it needs to be streamlined.

   Considering the additional analyses and explanations we have provided in the revision, that attempting to shorten the text will make the manuscript terse and harder to understand. PLoS Comp Biol does not have length limits and we hope that the reviewers and editors will accept the manuscript at its current length.

2. (9) Fig. 1: What are the units for the levels of RNA? Are they z-scores?

They are normalized microarray fluorescence intensities (May et al., 2013). We have indicated this in the figure legend.

3. (10) In Fig. 7, panels B, C, and D appear to be switched relative to the descriptions in the legend.

Fixed. Thanks!

4. (11) The link (p. 32) to the SPRING coordinates of cells in the Tusi et al. paper appears to be broken.

Fixed. Thanks!

## References


Vijay Chickarmane, Tariq Enver, and Carsten Peterson. Computational modeling of the hematopoietic erythroid-myeloid switch reveals insights into cooperativity, priming, and irreversibility. *PLoS Comput Biol*, 5(1):e1000268, Jan 2009. doi: 10.1371/journal.pcbi.1000268.

Richard Dahl, Jonathan C Walsh, David Lancki, Peter Laslo, Sangeeta R Iyer, Harinder Singh, and M Celeste Simon. Regulation of macrophage and neutrophil cell fates by the pu.1:c/ebpalpha ratio and granulocyte colony-stimulating factor. *Nat Immunol*, 4(10):1029–36, Oct 2003. doi: 10.1038/ni973.

R P DeKoter and H Singh. Regulation of b lymphocyte and macrophage development by graded expression of pu.1. *Science*, 288(5470):1439–41, May 2000.

Philipp S Hoppe, Michael Schwarzfischer, Dirk Loeffler, Konstantinos D Kokkaliaris, Oliver Hilsenbeck, Nadine Moritz, Max Endele, Adam Filipczyk, Adriana Gambardella, Nouraiz Ahmed, Martin Etzrodt, Daniel L Coutu, Michael A Rieger, Carsten Marr, Michael K Strasser, Bernhard Schauberger, Ingo Burtscher, Olga Ermakova, Antje Bürger, Heiko Lickert, Claus Nerlov, Fabian J Theis, and Timm Schroeder. Early myeloid lineage choice is not initiated by random pu.1 to gata1 protein ratios. *Nature*, 535(7611):299–302, 07 2016. doi: 10.1038/nature18320.

S. Huang, Y. Guo, G. May, and T. Enver. Bifurcation dynamics in lineage-commitment in bipotent progenitor cells. *Developmental Biology*, 305:695–713, 2007.

Graham D Jack, Li Zhang, and Alan D Friedman. M-csf elevates c-fos and phospho-c/ebpalpha(s21) via erk whereas g-csf stimulates shp2 phosphorylation in marrow progenitors to contribute to myeloid lineage specification. *Blood*, 114(10):2172–80, Sep 2009. doi: 10.1182/blood-2008-11-191536.

Peter Laslo, Chauncey J Spooner, Aryeh Warmflash, David W Lancki, Hyun-Jun Lee, Roger Sciammas, Benjamin N Gantner, Aaron R Dinner, and Harinder Singh. Multilineage transcriptional priming and determination of alternate hematopoietic cell fates. *Cell*, 126(4):755–66, Aug 2006. doi: 10.1016/j.cell.2006.06.052.

Gillian May, Shamit Soneji, Alex J Tipping, Jose Teles, Simon J McGowan, Mengchu Wu, Yanping Guo, Cristina Fugazza, John Brown, Göran Karlsson, Cristina Pina, Victor Olariu, Stephen Taylor, Daniel G

Tenen, Carsten Peterson, and Tariq Enver. Dynamic analysis of gene expression and genome-wide transcription factor binding during lineage specification of multipotent progenitors. *Cell Stem Cell*, 13(6): 754–68, Dec 2013. doi: 10.1016/j.stem.2013.09.003.

Noushine Mossadegh-Keller, Sandrine Sarrazin, Prashanth K Kandalla, Leon Espinosa, E Richard Stanley, Stephen L Nutt, Jordan Moore, and Michael H Sieweke. M-csf instructs myeloid lineage fate in single haematopoietic stem cells. *Nature*, 497(7448):239–43, May 2013. doi: 10.1038/nature12026.

Cristina Pina, Cristina Fugazza, Alex J Tipping, John Brown, Shamit Soneji, Jose Teles, Carsten Peterson, and Tariq Enver. Inferring rules of lineage commitment in haematopoiesis. *Nat Cell Biol*, 14(3):287–94, Mar 2012. doi: 10.1038/ncb2442.

Michael A Rieger, Philipp S Hoppe, Benjamin M Smejkal, Andrea C Eitelhuber, and Timm Schroeder. Hematopoietic cytokines can instruct lineage choice. *Science*, 325(5937):217–8, Jul 2009. doi: 10.1126/science.1171461.

Betsabeh Khoramian Tusi, Samuel L Wolock, Caleb Weinreb, Yung Hwang, Daniel Hidalgo, Rapolas Zilionis, Ari Waisman, Jun R Huh, Allon M Klein, and Merav Socolovsky. Population snapshots predict early haematopoietic and erythroid hierarchies. *Nature*, 555(7694):54–60, 03 2018. doi: 10.1038/nature25741.
