# Supplementary material for: Data-driven modeling predicts gene regulatory network dynamics during the differentiation of multipotential hematopoietic progenitors
Source: PLoS Comput Biol. 2022 Jan 14;18(1):e1009779. doi: 10.1371/journal.pcbi.1009779 (PMC8794271; doi:10.1371/journal.pcbi.1009779)
Supplement: Attachment — Submitted filename: grn_ery_neu_response_to_reviewers_rev1.pdf [file pcbi.1009779.s008.pdf]

Responses to reviewer comments on manuscript #PCOMPBIOL-D-21-01078R1

Overview: We thank the reviewers for their critical reading of the revised manuscript and detailed comments. We have further revised the manuscript in response and have addressed all of the comments, largely following the reviewers' advice. In particular, we have ascertained the statistical significance of *Spi1* upregulation along the neutrophil lineage in the Tusi *et al.* dataset as suggested by reviewer 1. Reviewer 2 disagreed with some of our claims and interpretations. We have revised our interpretations to be more conservative and circumspect than before. Specifically, we have reworded statements about *Spi1* not being the causal driver of neutrophil differentiation or being downstream of *Cebpa*. Those statements have been changed to focus on the activation of *Spi1* by *Cebpa* during the later stages of neutrophil development. A point by point description of the changes follows.

Major points

Reviewer 1

1. The reviewer suggests that misprediction of *Cebpa* and *Gfi1* in the PU.1ERT experiment could be the result of omitting macrophage genes from the model and would like us to discuss that.

    1. In previous reviewer comments, there was some concern about the interpretation of PU.1 effects, based on the different dose-dependent impacts of PU.1. In general, lower levels of PU.1 seem to be important in B cell development and neutrophil development, while higher levels of PU.1 drive macrophage/monocyte development. The authors have added several comments acknowledging that PU.1 effects are dose-dependent, but they do not really explore what this entails. In fact, this problem is also related to another reviewer concern, which was the neglect of any other fate choices that the cells may face besides the one between neutrophil and erythroid fates. This concern is directly relevant to the model predictions as compared to measurements shown here. Whereas B cell development is not accessible from the FDCP-mix model, there is good published evidence that the neutrophil (low PU.1) vs. macrophage (high PU.1) fate choices involve genuine mutual repressions between alternative sets of PU.1 collaborators, C/EBPalpha and Gfi1 on the one hand, and Irf8, potentially also Egr1 and 2, on the other hand (Laslo et al. 2006 Cell, Olsson et al 2016 Nature). Irf8 and the Egr factors are not included in the model, but it is at least possible that they are causing the otherwise paradoxical antagonisms seen when PU.1 levels are raised abnormally high, in the PU.1-ERT data in Fig. 3. If this explanation is correct, then the "conflict" between data and model predictions arises only from the simplifying assumptions in the model that disregard mechanisms involved in fate branches other than the neutrophil/ erythrocyte one.

    Obviously the authors should not have to make a completely new model at this point, especially when there is no comparable time series evidence to use for optimization. But it is worth making it clearer that the dose dependences of factor actions (presumably for Gfi1 as well as for PU.1) can lead to alternative pathways, and that the regulators of these alternative pathways are not all included in the model. Also, the data showing PU.1-ERT

activation shutting down the neutrophil program is not an anomaly, but exactly what would have been predicted based on evidence from Laslo et al., 2006 (Cell), and Kueh et al., 2013 (Science), although those were not whole-genome studies.

Good point! We now discuss this in paragraph 2 on p. 14.

2. The reviewer would like us to explain/clarify modeling choices about baseline transcription and when genes are interpreted as being repressed vs activated.

> 2. The choice of baseline values in the modeling is somewhat surprising. As noted in passing in the text (section 2.4), the model (section 4.1) structures the sigmoidal regulation-expression level function S(u) such that when regulatory input is zero, the value of S is 0.5. Since values <0 are allowed in the model, it is not clear why baseline transcription is arbitrarily set at half maximal.
>
> a. This is a peculiar choice for a mammalian system, where most would consider that the default for a gene with zero regulatory input would be no expression. Please explain.
>
> b. It also leads the authors to interpret values of expression between 0 and 0.5 x maximal (in Fig. 6, at time 0) as evidence that genes are actually undergoing repression (p. 17), whereas most would interpret values >0 as evidence of weak activation. The authors' interpretation of multilineage priming thus becomes the exact opposite of what others in the field have thought, even though based on the same data.

a. Baseline transcription in the model is not half max, ie $u = 0$, but $u = h_i$. This can be seen by setting the concentrations of all regulators to zero in the expression for u (paragraph 1 on p. 6). The value of h_i is not predetermined, but is determined by fitting to data. As it happens, h_i has been inferred to be negative for all the genes (Table S1) and therefore the baseline expression of the genes is something less than half-max. The fact that h_i determines the baseline expression was mentioned in the third last sentence of the same paragraph. We are not explaining this further since there is quite an extensive gene circuit literature where this is explained in detail.

b. There are two senses in which the word "repressed" could be used. The first is to describe the expression level of a gene. The second sense is that a gene is repressed is when repressors (TFs) cause a reduction in the rate of transcription of the gene.

It is the second usage that we meant to employ on p. 17 of revision 1 while describing Fig. 6

> All the genes are in a partially repressed state, since their total regulatory input is negative, in undifferentiated FDCP-mix cells. This is reminiscent of multilineage transcriptional priming [3, 60, 61]-the low-level expression of genes from multiple lineages in multipotential progenitors. What accounts for the repression varies by the target gene. Genes downregulated in neutrophil conditions, Gata1, Zfpm1, Klf1, Tal1, and Epor, are repressed by several genes of small effect. Genes downregulated in erythrocyte conditions however, Spi1, Cebpa, Gfi1, Stat3, Gata2, Il3ra, and Csf3r, are mainly repressed by a combination of Zfpm1 and Tal1.

but we see now that the first sentence implies that repression is inferred based solely on the fact that $u < 0$. We have clarified in paragraph 2 on p. 18 that the model infers repression in undifferentiated cells based on the predominance of the negative components of u (from repressors; above the dotted

line). In other words, the low level of transcription is due to the co-expression of repressors from the alternative lineage in addition to relatively low concentrations of activators.

Repressors need not act only in an all-or-none manner. Many studies demonstrate the dependence of reporter expression on the concentration/dose of repressors (see Laslo et al., 2006, for example). This happens at a single-cell level; perhaps the best demonstration comes from the *Drosophila* blastoderm. Repressors do not shut off transcription completely but permit low or intermediate levels of expression of the target gene (see Clyde et al., 2003, for example) in a concentration-dependent manner.

Co-expression at a single-cell level and mutual repression is the basis of the bistable switch model, and hence our interpretation of multilineage priming is consistent with prior literature. For example Chickarmane et al. (2009) say:

> In [33], using a combination of mathematical modeling and experimental data, it is shown how the PU.1–GATA-1 network encodes the possibility of priming. Assuming that PU.1 and GATA-1 repress each other's expression, the only possible states of the system are either PU.1 at a high level and GATA-1 at a low level, and vice versa, or an unstable "progenitor" state at which both PU.1 and GATA-1 are expressed at intermediate levels.

In other words, in progenitors, GATA1 and PU.1 are expressed at intermediate levels as a result of their mutual repression.

3. The reviewer would like us to test whether the upregulation of *Spi1* in the Tusi *et al.* data is statistically significant.

> 3. The validation of late Spi1 upregulation in data from Tusi et al. is suggestive, but not overwhelming. While this could be due to technical difficulties in the single-cell RNA-seq analysis, it might be valuable to calculate the statistical significance of the differences in Fig. 7B between Spi1 levels at the peaks of Cebpa and Gfi1 expression (potential 13.7-9.7) and after those factors are downregulated (potential >100). Also, with regard to the first point, is there any comparison that can be made between these levels of Spi1 and the levels in the Monocyte/Macrophage branches of the SPRING plot?

We have performed Welch's two-sample t-test comparing *Spi1* levels in the bins with differentiation potential 13.7 (*Cebpa* peak) and 9.7 (*Gfi1* peak) to the bin with differentiation potential -109 (penultimate bin). The p-values were 0.004 and 0.04 respectively. Described in para 2 on p. 21.

We also compared *Spi1* levels between the macrophage and neutrophil lineages in Tusi *et al.*'s data as suggested by the reviewer. Comparisons between lineages in the SPRING plots are complicated by the fact that the number of cells in each lineage differ widely. There are $\sim$1,700 cells accorded more than 50% probability of developing into granulocytes, while there are only 300 such cells for the macrophage lineage. Furthermore, the PBA potential also differs between the lineages, ranging from -141 to 22 in the granulocyte arm and from 18 to 22 in the monocyte arm. This makes it unclear what an apples to apples comparison would be. We compared all the cells having greater than 50% chance in granulocyte vs. monocyte lineages and found that the mean expression was 0.8 in the former and 0.66 in the latter ($p = 0.02$). If we restrict the granulocyte PBA potential range to be same as monocyte one (18-22), the mean expression in the granulocyte arm reduces to 0.62 ($p = 0.5$). We have not included this comparison since the monocyte lineage is out of the scope of the manuscript.

Reviewer 2

1. The reviewer asserts that the activation of *Spi1* by *Cebpa* demonstrated here is limited to the maturation of neutrophils—but not in cell-fate choice—since *Spi1* is upregulated later in FDCP-mix differentiation. Secondly, the reviewer is unconvinced by our argumentation that *Spi1* is not the causative agent for neutrophil specification. The reviewer would like us to revise our claims that *Spi1* is not the causative agent of neutrophil differentiation.

    > (5) The manuscript should clearly distinguish between lineage-choice (cell fate decisions), which is often referred to as cell differentiation, and cell maturation, which is the acquisition of the characteristics of mature, circulating blood cells after lineage choice. The increase in levels of Spi1 mRNA that is the focus of much of the presentation occurs late in the time-course, long after lineage choice. The manuscript could describe the inferred role of SPI1 as a "hub" (terminology from page 23) to ensure expression of neutrophil-specific genes during the later time points (which themselves are proxies for the later stages of maturation). However, the claims that SPI1 is "not the causative agent of neutrophil development" (p. 3), "Cebpa and Gfi1 are upstream of Spi1 in the genetic architecture of neutrophil differentiation" (page 18), "Spi1 is downstream of Cebpa and Gfi1 in the chain of causation leading to the specification of neutrophils" (page 20), "insight that Cebpa and Gfi1 and not Spi1 are the causal drivers of neutrophil differentiation" (p. 22), and elsewhere are not correct. Those claims could be couched in terms of CEBPA being implicated in the further up-regulation of Spi1 gene expression late in the time course of FDCP-mix cell differentiation, which is a proxy for later stages of neutrophil maturation. Importantly, the data and modeling do NOT argue against a role for SPI1 in neutrophil specification.

    We agree with the reviewer that the late upregulation of *Spi1* suggests that *Spi1* is involved in the maturation of neutrophils. However, a stronger claim can be made.

    FDCP-mix cells retain the potential for multiple lineages in IL3 media, implying that lineage choice is made after GCSF treatment. The choice must be getting made during the early stages of the differentiation. In fact, May et al. (2013) showed that FDCP-mix cells lose self-renewal ability in Epo conditions between 24 and 48 hours. This implies that the set of factors involved in causally making the choice must change expression during the earlier stages. The fact that a gene is expressed is not sufficient to guarantee a role in cell-fate specification—its expression must change. The facts that *Spi1* 1) acts in a concentration-dependent manner, 2) stays at a constant level during the first 2 days of FDCP-mix differentiation, and 3) is only upregulated in the later stages implies that it is not in the set of factors choosing the neutrophil fate in FDCP-mix cells. That is not to say *Spi1* does not play a role in the lineage decision - it is expressed in undifferentiated FDCP-mix cells and must be contributing to the regulation of neutrophil specification genes. Another way to put this is that mere *Spi1* expression in undifferentiated FDCP-mix cells is necessary but not sufficient for neutrophil differentiation. It must not be sufficient, otherwise FDCP-mix cells would spontaneously differentiate into neutrophils.

    The same may not be claimed about the *in vivo* situation since we don't know whether the cells having greater than 50% chance of becoming neutrophils according to the PBA algorithm (Fig. 7) have already chosen the fate or not. Accordingly, we had merely speculated that inferences about FDCP-mix cells could apply to the *in vivo* situation.

Given that these arguments and interpretations involve a certain level of subjectivity, we have followed the reviewer's advice and made our claims more circumspect. We have rephrased them so that we no longer claim that *Spi1* is not the causative agent of neutrophil differentiation nor that it is downstream of *Cebpa*. We limit our claims to the role *Cebpa* plays in the upregulation of *Spi1* during the latter half of FDCP-mix neutrophil differentiation. As discussed above, treating FDCP-mix cells with GCSF involves both choosing a cell fate as well as maturation and the two are not easily separated. Therefore we use the term "neutrophil development". The language has been adjusted in all the instances pointed out by the reviewer. We have also made such changes elsewhere in the manuscript, including the abstract, the last paragraph of the introduction, and the results.

The list of changes:

(a) Sentence 9 of abstract: implication that the bistable switch does not drive differentiation has been removed and replaced with statement that other regulatory influences (Cebpa) outside of the bistable switch cause later PU.1 upregulation.

(b) paragraph 2 on p. 19: removed sentence that Cebpa is upstream of Spi1. Changed "differentiation" to "development".

(c) paragraph 2 on p. 21: rephrased statement about Cebpa being upstream of Spi1.

(d) paragraph 1 on p. 22: rephrased last sentence about Spi1 being downstream of Cebpa.

(e) paragraph 2 on p. 23: rephrased first sentence about Spi1 not being a causal driver of neutrophil differentiation. Removed last sentence stating that neutrophil differentiation is caused by the upregulation of Spi1 by Cebpa and Gfi1.

(f) paragraph 3 on p. 23 has been reworked to focus on the later upregulation of Spi1 and to remove claims that Spi1 is downstream of Cebpa etc.

Please note that we have retained our discussion of Hoppe *et al.*'s (Hoppe et al., 2016) live cell tracking experiments and their implication that the Gata1-PU.1 bistable switch does not initiate cell-fate choice between the erythroid and myeloid fates (paragraph 2 on p. 23). This is a peer-reviewed published study and it is appropriate to discuss those interpretations and their connection to our work even if some may not agree with them.

2. The reviewer presents two arguments that our claim that Spi1 is not the initiator of neutrophil differentiation is incorrect. First, the reviewer points out that Spi1 is expressed at ∼25% of its peak expression in undifferentiated FDCP-mix cells, the Tusi *et al.* data, and other databases and that level is substantial and therefore implies that *Spi1* plays a role in neutrophil differentiation. Second, the reviewer argues that mutual antagonism between PU.1 and Gata1 implies that PU.1 is an initiator.

> (4) The manuscript emphasizes that the gene circuit models argue against SPI1 (PU.1) being involved in initiation of the neutrophil cell fate decision, and ascribe that role to CEBPA and GFI1. This conclusion is largely derived from an increase in level of Spi1 mRNA in the later stages of neutrophil "differentiation", while the levels of this mRNA earlier in the time course are considered to be insufficient for SPI1 to play a role in determining the neutrophil cell fate. However, if the scales for mRNA levels refer to "fraction of maximum", then the levels of Spi1 mRNA at early time points appear to be about 25-30the maximum, which would be a rather high level. Data from single cell transcriptomes (Fig. S4 and Fig. 7) also show a substantial level of Spi1 mRNA in progenitors, which is also clear in

many other transcriptome datasets on hematopoiesis (e.g. from BloodSpot database and servers). These levels of SPI1 are sufficient for this protein to play a role in lineage choice. Examination of Fig. 7 reveals a pattern that is consistent with SPI1 playing an early role in lineage choice. Fig. 7A shows cells with a high probability of differentiating to neutrophils as green, which covers the entire right "leg" of the display. Those cells all have notable expression of the Spi1 gene (as well as Cebpa and Gfi1 genes) denoted as yellow. Thus, the Spi1 gene is expressed at a stage at which it could play a role in lineage choice. The revised manuscript does include a statement on page 23 acknowledging the mutual antagonism between SPI1 and GATA1 in cell fate decision, but the next paragraph argues that the Spi1 gene does not have an initiator role in differentiation. These assertions seem to be counter to each other.

Here, as in the previous point, the reviewer disagrees with our interpretation of the data and modeling results with regards to the role *Spi1* plays in neutrophil differentiation. Due to the subjectivity inherent in interpretations, we have limited our claims to the role *Cebpa* and, possibly *Gfi1*, play in upregulating *Spi1* during FDCP-mix neutrophil development as suggested by the reviewer (see our response to reviewer point #5 above).

However, we would like to point out that 1) a gene's (robust or otherwise) expression in progenitor cells is by itself no guarantee of having any role, leave alone that of an initiator, in differentiation. For factors such as PU.1 that act in a concentration dependent manner, a necessary condition for a causal or initiating role is that their expression *change* during differentiation. 2) There is a difference between "having a role" and "causing" or "initiating" differentiation. Certainly, *Spi1* is expressed in the progenitor cells, and clearly has a role in specifying the neutrophil fate. However, it is unlikely to be an early responder to GCSF signaling in FDCP-mix cells since it does not change expression until 2 days have elapsed. 3) The reviewer asserts that antagonism between *Spi1* and *Gata1* implies that *Spi1* has an initiator role in differentiation. The antagonism between the factors does not preclude the possibility that one or both factors respond to outside signals that alter their balance. In other words, the bistable switch need not be isolated. As we had explained in our previous response, bistable switch models include a factor "X" that serves the purpose of tipping the balance in favor of one fate or the other (Huang et al., 2007; Chickarmane et al., 2009). In other words, the bistable switch is embedded in a larger gene regulatory network.

3. The reviewer thinks it is better if the gene circuits equations and their explanation is introduced early in the results instead of the Methods.

(1) The revised version does clarify the differences between the gene circuit models and previous ones. However, the manuscript was not revised the to answer "Exactly what is the output of the gene circuit analysis?" It turns out that the output is the table of interaction coefficients and other parameters. However, those coefficients and parameters are not explained until section 4 (Materials and Methods). It would be more effective and clear for readers to have equation (1) with the explanation of the terms introduced early in the Results.

We have done so and added a statement further explaining that the genetic interconnectivity coefficients are one of the most important outputs of the modeling procedure:

Training gene circuit models on quantitative gene expression data results in estimates of the values of these parameters. Estimates of the genetic inteconnectivity coefficients (T_{ij}) allows the inference of the genetic architecture of the GRN.

4. The reviewer asks us to qualify our claims as applicable only to the FDCP-mix system.

   (2) The manuscript should focus the statements about accuracy and predictions primarily to the FDCP-mix differentiation experiment, and be cautious about conclusions with regard to neutrophil differentiation. The revised manuscript does point out that levels of Gata2 gene expression are much higher in the FDCP-mix experiment than is seen during normal neutrophil differentiation. However, despite the utility FDCP-mix cells as models for several studies, they are not the same as primary, multi-lineage progenitor cells.

   We have added qualifiers to statements indicating that they pertain to FDCP-mix cells:

   (a) paragraph 3, p. 21: third last sentence
   (b) paragraph 5, p. 18: fourth sentence
   (c) Title of section 2.4
   (d) paragraph 3, p. 16: first sentence
   (e) Title of section 2.1.5

5. The reviewer would like us to clarify what the units in the gene expression plots.

   (3) The revision did not clarify what values are being graphed in Figure 1 and other figures, but given the values appear to range from 0 to 1.0, and the axis is labeled "Relative expression", perhaps these measurements are fractions of the maximum level observed over the time course. If so, it would help readers to state that explicitly. Perhaps that is what "normalized" in the legend refers to, but normalization can done in many different ways. Knowing clearly what the units are is important, as discussed in the next point.

   Although we had explained the normalization in Methods in Section 4.2, we had erroneously stated in the response that this explanation is contained in the figure legend. We apologize for this confusion. We have included the explanation of the units/relative expression in the legend of Fig. 1 now. May *et al.*'s microarray flourescence intensities, which had already been normalized across samples, were scaled so that the maximum intensity across all samples was 1.

6. The reviewer asks us to explain how the discrepancy in Gata2 expression between FDCP-mix and bone marrow cells could result in mispredictions in FDCP-mix cells.

   (6) As mentioned above, the increase in expression of Gata2 in neutrophils observed in the FDCP-mix transcriptome data of May et al. is not observed in primary cells, as now stated in the revised manuscript (p. 11). The revised manuscript further states that this difference may account for the discrepancy between prediction and observation of the effects of Gata2 knockdown in FDCP-mix cells (Fig. 3 and text on p. 11). However, the model in the manuscript was trained on data from FDCP-mix cells, so the predictions should still be accurate with respect to FDCP-mix, but not with respect to primary cells. The lack

> of correspondence between the Gata2 RNA data in FDCP-mix cells and primary cells is important to present and discuss, but it would not appear to account for the discrepancy between simulation and observation in FDCP-mix cells. If indeed there is a strong logic for the ability to account for the discrepancy, the manuscript should elaborate on it.

This discrepancy impacts the model's inferences and predictions indirectly. The model is unable to properly infer and predict *Gata2* related parameters and perturbations respectively because *Gata2* is upregulated in both conditions. This expression pattern does not bear much information about *Gata2*'s regulation. The lack of differential expression between conditions is a consequence of the discrepancy between FDCP-mix and BM expression.

We have changed the paragraph (para 2 on p. 12) and discussion (para 3 on p. 25) to limit our reasoning to the direct reason, namely the similarity of *Gata2* expression between the two conditions. The discussion of the discrepancy in *Gata2* expression between FDCP-mix cells and BM has been moved up to the paragraph that describes the expression patterns (para 3, p. 8). The supplementary figures have been renumbered to reflect the order in which they are discussed in the revised manuscript.

Other points

Reviewer 1

1. 4. Minor point: in Fig. 7, please change the color of the "zero detected" dots, or else add a note that a black dot in Fig. 7A and D represents failure of detection, not maximal expression.

   We have added an explanation for the black dots in Fig. 7A,D in the legend.

2. 5. Minor point: The term "data-driven" is applied too narrowly. There is an implication that only whole-genome, bulk population observational studies are a valid source for "data". But gene network models can establish quite a few biologically important connections using data from other kinds of experiments, especially experiments involving deliberate, focused perturbations that are possible in model systems.

   This impression was unintended. We have clarified in paragraph 3 on p. 4 (fourth sentence).

Reviewer 2

1. (7) p. 14: After the manuscript presents cases of agreement and conflict between the predictions and experimental result, an overly positive conclusion is drawn: "These inconsistencies were, however, a small proportion of the total inferences and the overall good agreement between model inference and empirical evidence (Fig. 4 and Table S1) suggests a successful decoding of the genetic architecture." A more accurate assessment would be that "...the overall good agreement between model inference and empirical evidence (Fig. 4 and Table S1) suggests a *partially* successful decoding of the genetic architecture."

   We have removed the claim about successfully decoding the genetic architecture (paragraph 2, p. 15).

2. 8) p. 18: The manuscript concludes that the "activation of neutrophil targets by Gfi1 inferred by the model could, in fact, represent the activity of C/EBPalpha", but elsewhere, including the Abstract and Discussion, that activation is stated as resulting from the input of both proteins. That inconsistency should be corrected.

The apparent inconsistency arises from the fact that the model predicts that *Spi1* is activated by *Cebpa* and *Gfi1*, while the sentence in question is pointing out that the model may not necessarily be able to distinguish between *Cebpa* and *Gfi1* effectively. It is also possible however that *Gfi1* is activating *Spi1* indirectly as discussed in sentence 6 in the same paragraph (paragraph 5, p. 18):

> The activation role inferred here for Gfi1 during neutrophil differentiation could result from indirect regulation of its targets.

Given the former possibility, we had qualified the inference that *Gfi1* upregulates neutrophil genes.

We have addressed this inconsistency by explictly distinguishing between model prediction and its qualified interpretation (sentence 5 of the abstract and paragraphs 1–3 of discussion).

3. (9) Top of page 26: The summary conclusion at the end of the Discussion paragraph on inaccuracies in inferences about GATA2 should state clearly that those inferences are incorrect with respect to differentiation in primary cells, not just that users should be "careful about" those inferences.

Although the entire paragraph was dedicated to discussing how the *Gata2* related inferences were incorrect, we have removed the last sentence about being careful about interpreting *Gata2* inferences and added the following phrase to the topic sentence: "... implying that *Gata2*-related inferences are incorrect for both FDCP-mix and *in vivo* differentiation." (paragraph 3, p. 25)

4. (10) The labels for panels B, C, and D in Fig. 7 are in a different order from the description in the legend, but it is the same as in the text.

The legend has been fixed. Thanks.

5. (11) The text is overly long, and it needs to be streamlined.

Considering the additional analyses and explanations we have provided in the two revisions, that attempting to shorten the text will make the manuscript terse and harder to understand. PLoS Comp Biol does not have length limits and we hope that the reviewers and editors will accept the manuscript at its current length.

References

Vijay Chickarmane, Tariq Enver, and Carsten Peterson. Computational modeling of the hematopoietic erythroid-myeloid switch reveals insights into cooperativity, priming, and irreversibility. *PLoS Comput Biol*, 5(1):e1000268, Jan 2009. doi: 10.1371/journal.pcbi.1000268.

D. E. Clyde, M. S. Corado, X. Wu, A. Pare, D. Papatsenko, and S. Small. A self-organizing system of repressor gradients establishes segmental complexity in *Drosophila*. *Nature*, 426:849–853, 2003.

Philipp S Hoppe, Michael Schwarzfischer, Dirk Loeffler, Konstantinos D Kokkaliaris, Oliver Hilsenbeck, Nadine Moritz, Max Endele, Adam Filipczyk, Adriana Gambardella, Nouraiz Ahmed, Martin Etzrodt, Daniel L Coutu, Michael A Rieger, Carsten Marr, Michael K Strasser, Bernhard Schauberger, Ingo Burtscher, Olga Ermakova, Antje Bürger, Heiko Lickert, Claus Nerlov, Fabian J Theis, and Timm Schroeder. Early myeloid lineage choice is not initiated by random pu.1 to gata1 protein ratios. *Nature*, 535(7611):299–302, 07 2016. doi: 10.1038/nature18320.

S. Huang, Y. Guo, G. May, and T. Enver. Bifurcation dynamics in lineage-commitment in bipotent progenitor cells. *Developmental Biology*, 305:695–713, 2007.

Peter Laslo, Chauncey J Spooner, Aryeh Warmflash, David W Lancki, Hyun-Jun Lee, Roger Sciammas, Benjamin N Gantner, Aaron R Dinner, and Harinder Singh. Multilineage transcriptional priming and determination of alternate hematopoietic cell fates. *Cell*, 126(4):755–66, Aug 2006. doi: 10.1016/j.cell.2006.06.052.

Gillian May, Shamit Soneji, Alex J Tipping, Jose Teles, Simon J McGowan, Mengchu Wu, Yanping Guo, Cristina Fugazza, John Brown, Göran Karlsson, Cristina Pina, Victor Olariu, Stephen Taylor, Daniel G Tenen, Carsten Peterson, and Tariq Enver. Dynamic analysis of gene expression and genome-wide transcription factor binding during lineage specification of multipotent progenitors. *Cell Stem Cell*, 13(6): 754–68, Dec 2013. doi: 10.1016/j.stem.2013.09.003.
